# Short- and Long-Term Outcomes in Elderly Patients with Resectable Esophageal Cancer: Upfront Esophagectomy Compared to Surgery after Neoadjuvant Treatments

**DOI:** 10.3390/jcm13144271

**Published:** 2024-07-22

**Authors:** Lucia Moletta, Elisa Sefora Pierobon, Giovanni Capovilla, Irene Sole Zuin, Jose Luis Carrillo Lizarazo, Giulia Nezi, Sara Lonardi, Sabina Murgioni, Sara Galuppo, Gianpietro Zanchettin, Renato Salvador, Luca Provenzano, Michele Valmasoni

**Affiliations:** 1 1st Surgical Clinic, Department of Surgery, Oncology and Gastroenterology, University of Padova, 35128 Padova, Italy; 2 Department of Oncology, Veneto Institute of Oncology IOV—IRCCS, 35128 Padova, Italy; 3 Radiotherapy Unit, Veneto Institute of Oncology IOV—IRCCS, 35128 Padova, Italy

**Keywords:** esophageal cancer, elderly, frailty, upfront esophagectomy, multimodal treatment

## Abstract

**Background/Objectives:** Despite the increased incidence of esophageal cancer (EC) in elderly people, there are no clear guidelines for its treatment in these patients. The aim of this study was to compare the outcomes of patients ≥ 75 years with resectable EC, receiving either upfront esophagectomy or neoadjuvant treatment. **Methods**: We retrospectively identified 127 patients with resectable EC ≥ 75 years who underwent esophagectomy between January 2000 and December 2022 at our Clinic in the University Hospital of Padova. The included patients were stratified into two groups: patients undergoing upfront esophagectomy (SURG group) and patients receiving neoadjuvant treatment (NAT group). **Results**: There were no statistically significant differences in OS (*p* = 0.7708), DFS (*p* = 0.7827) and cancer-related survival (*p* = 0.0827) between the SURG and the NAT group, except for the OS of EAC with stage III-IV, where the NAT group experienced a significant benefit in OS (*p* = 0.0263). When comparing the two groups, patients receiving neoadjuvant treatment experienced a significantly higher rate of postoperative complications (*p* = 0.0266). At univariate analysis, neoadjuvant therapy was the only variable strongly associated with postoperative morbidity (*p* = 0.026). **Conclusions:** Considering the unique characteristics of elderly patients, the choice of a multimodal approach should be tailored to each case in a multidisciplinary setting and balanced with a potential higher risk of postoperative complications, as well as potential toxicity related to chemoradiation and reduced life expectancy.

## 1. Introduction

According to GLOBOCAN 2022, esophageal cancer (EC) is the 11th most common malignant tumor for both sexes in terms of incidence, with approximately 511,000 new cases in 2022 globally [1]. It is the seventh leading cause of cancer-related death worldwide, with approximately 445,391 deaths per year [1]. As its mortality rate is almost equal to its incidence rate, the prognosis for this cancer is clearly poor, with a 5-year survival rate as low as 20% [2,3]. The change in life expectancy in recent decades has led to neoplasms of the digestive tract in the elderly becoming a major issue in the public health system [4]. In fact, the median age of patients with esophageal and gastroesophageal junction cancer is 68 years, with approximately 30% of patients older than 75 years and 8% over 85 years old when diagnosed [3]. Despite the increased incidence of EC in elderly people, there are no clear guidelines for its treatment in these patients [5,6]. Moreover, outcomes of older patients with locally advanced EC are not well characterized, as these patients are frequently underrepresented in clinical trials [7,8]. Although the mainstay of treatment for locally advanced EC is preoperative chemoradiation followed by esophagectomy [9], there are valid concerns regarding such an aggressive multimodal approach in elderly people. Compared to younger patients, older patients have more comorbid conditions, higher risk of postoperative complications, and are more prone to the potential toxicity of chemoradiotherapy. Furthermore, given the intrinsically shorter life expectancy, it is not clear whether a multimodal approach truly confers a survival benefit in elderly people. Finally, older patients may receive reduced dose adjustments in light of their comorbidities or frailty, possibly affecting their overall survival. Only a few studies have addressed the postoperative outcomes of elderly people according to the therapeutic approach they received. The aim of this study was to compare the short- and long-term outcomes of patients ≥ 75 years with resectable EC receiving either upfront esophagectomy or neoadjuvant treatment before surgery in order to evaluate the feasibility of a multimodality preoperative treatment in these patients.

## 2. Materials and Methods

### 2.1. Cohort Selection

We retrospectively identified patients with resectable EC ≥ 75 years who underwent esophagectomy between January 2000 and December 2022 at our clinic in the University Hospital of Padova, from a prospectively collected database. Inclusion criteria were: resectable adenocarcinoma (EAC) or squamous cell carcinoma (SCC) of the thoracic esophagus or the esophagogastric junction (EGJ) in patients ≥ 75 years undergoing esophagectomy with curative intent, with or without neoadjuvant therapy before surgery. We chose an age threshold of 75 years, because it was deemed most relevant for current surgical practice, and prominent randomized controlled trials (TIME, MIRO) used this age threshold as an exclusion criterion [10,11]. Patients receiving palliative surgical procedures, undergoing salvage esophagectomy or with rare histology were excluded from the study. Additionally, we excluded patients with tumors located in the cervical esophagus, given the different therapeutic approach usually reserved for these cases. Patients were also excluded if they received only chemoradiotherapy after being deemed unfit for surgery and if there were missing surgical and survival data. The included patients were stratified into two groups: patients undergoing upfront esophagectomy (SURG group) and patients receiving neoadjuvant treatment before surgery (NAT group). Preoperative and postoperative data were compared between the two groups, as well as overall survival (OS) and disease-free survival (DSF).

### 2.2. Pretreatment Work-Up

All patients underwent esophagogastroscopy and biopsy to obtain histological diagnosis and assess tumor location and length. Furthermore, tumors were staged with computed tomography (CT), endoscopic ultrasound (EUS) and, in selected cases, with 18-FDG PET. The staging of esophageal cancer was defined according to the 8th edition of the TNM criteria proposed by the Union for International Cancer Control (UICC) [12].

Comorbidities and fitness of patients before surgery were classified according to the Charlson Comorbidity Index (CCI), the American Society of Anesthesiologists grade (ASA), and Karnofsky Performance Status.

All cases were discussed at the Tumor Board meeting for esophago-gastric cancer at our center. The decision between neoadjuvant therapy plus surgery or upfront surgery was tailored for each patient considering the characteristics and extent of the cancer, the characteristics and comorbidities of the patients, and the informed decision of the patient. Patients with tumors staged as T4b or patients with any T stage but with distant metastases were considered non-resectable. Neoadjuvant treatment varied according to the regimens available at the time of diagnosis. Patients with stage disease that could be eligible for neoadjuvant therapy (clinical stages greater or equal to T2N0 with risk factors or more advanced stages, excluding metastatic disease) who had contraindications to chemotherapy and chemoradiation underwent primary surgical resection.

### 2.3. Surgery

Surgical techniques included Ivor–Lewis esophagectomy for mid-lower lesions and McKeown esophagectomy for tumors located in the upper thorax. The preferred reconstruction modality was with a gastric conduit, followed by the middle-left colon or the jejunum. We routinely performed a standard two-field lymphadenectomy, extended to a three-field lymphadenectomy in the case of suspected cervical lymph node involvement. Minimally invasive esophagectomy was performed when feasible.

Postoperative 90-day complications were classified according to the Esophagectomy Complications Consensus Group [13], and the severity of each complication was labeled according to the Clavien–Dindo classification [14]. Perioperative mortality was considered as death within 90 days of surgery.

### 2.4. Follow-Up and Outcome

Patients were followed up clinically and radiologically after surgery every 3 months for the first year, every 6 months for the following 4 years, and then annually. Overall survival (OS) was calculated from the date of surgery until the date of death or last follow-up. Disease-free survival (DFS) was calculated from the date of diagnosis until recurrence, last follow-up or the date of death.

### 2.5. Statistical Analysis

The whole study cohort was analyzed and subset analyses of the following groups were performed: patients undergoing upfront surgery (SURG group) and patients undergoing neoadjuvant therapy before surgery (NAT group). Descriptive results are shown as mean ± standard deviation for continuous variables and as size and frequency for categorical variables. Continuous variables not normally distributed are reported as median and interquartile ranges. Quantitative variables were compared using the Mann–Whitney test or the Kruskal–Wallis test, as appropriate. Categorical variables were analyzed with the chi-square test or Fisher’s exact test in the case of absolute frequencies < 5. Univariate analysis was performed to identify possible risk factors for postoperative morbidity. Variables showing a *p*-value < 0.1 on univariate analysis were evaluated by multivariate logistic regression, to identify independently associated risk factors for these outcomes. The threshold for statistical significance was established at a *p*-value less than 0.05.

Overall and recurrence-free survival estimates were calculated using the Kaplan–Meier method. The survival curves were compared using the log rank test or the Gehan-Breslow-Wilcoxon test, as appropriate. DFS was defined as the time from the surgical procedure to disease recurrence or death, regardless of the cause. The diagnosis of disease-recurrence during follow-up required confirmation of pathological PET-CT uptake of newly identified lesions at follow up CT scan and/or histological confirmation of lesions amenable to biopsy. Locoregional recurrences were defined as those occurring at the anastomotic or perianastomotic region of the gastric conduit or esophageal remnant and those involving the lymph nodes included in the two-field lymphadenectomy. Distant recurrences were defined as the presence of disease in outfield lymph nodes (cervical, supraclavicular, or below the level of the pancreas), peritoneal carcinomatosis, malignant pleural effusions or metastases to distant organs. OS was defined as the time from the surgical procedure to death, regardless of the cause. Disease-specific mortality was defined as death occurring as a consequence of locoregional or distant disease relapse.

All statistical analyses were performed using JMP software version 12.0.1 for MacOS (SAS Institute Inc., Cary, NC, USA) and GraphPad Prism software version 8.4.3 for MacOS (San Diego, CA, USA).

All procedures were carried out according to our research and ethical guidelines. This study was approved by the Research Committee of the Department of Surgical, Oncological, and Gastroenterological Sciences at the University of Padova. All patients provided their written consent for the use of their data for research purposes.

## 3. Results

From 2000 to 2022, 2197 patients underwent esophageal resection for cancer at our Center. Among these, a total of 127 patients met the inclusion criteria and were included in the study. Seventy-eight patients underwent upfront esophagectomy (SURG group, 61.4%) and 49 were treated with neoadjuvant therapy before surgery (NAT group, 38.6%).

### 3.1. Preoperative Data

Demographic data are summarized in Table 1.

The median age of the entire cohort was 78 years (76–80) with 45 patients aged over 80 years. No significant differences were observed in comorbidities, overall patient health, and symptoms between the two groups. However, patients in the SURG group demonstrated a higher frequency of elevated ASA scores (*p* = 0.0371). No significant differences were observed in the location of the tumor between the two groups. SCC histology was more frequent in the NAT group, while EAC was more frequent in the SURG patients (*p* = 0.0005). Regarding the initial clinical stage of the tumor, patients who underwent NAT tended to have tumors with a higher T stage (*p* = 0.0006) and a higher number of metastatic lymph nodes (*p* < 0.0001) when compared to patients who underwent initial surgery. Patients undergoing NAT had a higher clinical stage compared to patients undergoing upfront surgery (*p* < 0.0001). In particular, earlier clinical stages were more represented in the SURG group, while stages III-IV were more frequent in the NAT group (*p* < 0.0001).

### 3.2. Neoadjuvant Treatment

The neoadjuvant treatment was not standardized as patients were commonly referred for surgery after being treated at other centers; therefore, potential variations in the selected treatment regimens were subject to the discretion of attending oncologists or influenced by the individual clinical profiles and comorbidities of the patients. Among patients receiving NAT before surgery, 28 patients were treated with a platinum-based regimen (57.14%), 12 with a taxane-based regimen (24.5%) and 9 with other regimens (18.4%). Thirty-two patients received concurrent chemoradiotherapy (65.3%). Almost half of the patients (*n* = 21, 47.7%) experienced adverse events during chemotherapy or chemoradiotherapy, leading to discontinuation of treatment in 5 cases (11.36%). The most frequent organ-related adverse events were hematological (*n* = 13, 29.54%), gastrointestinal (*n* = 4, 9.09%) and pulmonary (*n* = 2, 4.54%). The incidence of postoperative complications was 57% in the chemotherapy group and 62.8% in the chemoradiation group. No significant differences were detected between the two groups of patients, neither in the overall postoperative morbidity rate nor in the occurrence of specific postoperative complications.

### 3.3. Operative Data

No significant differences were observed in the two groups regarding the surgical approach (Table 2); Ivor-Lewis esophagectomy was the most common operation in both groups, followed by McKeown esophagectomy. Additionally, 5 underwent transhiatal esophagectomy and 6 underwent pharyngocoloplasty. No differences in median surgery duration were observed between SURG (281 [200–398] min) and NAT group (307 [210–401] min), as well as in estimated intraoperative blood loss (530 [200–820] mL vs. 540 [210–850] mL). Postoperative morbidity rate was 48.8%. Twelve patients required reoperation either for severe anastomotic leaks with hemodynamic instability due to sepsis, failure of conservative management or for the presence of diffuse ischemia or necrotic tissue. These cases required a rethoracotomy, anastomosis takedown, and gastric tube/coloplasty resection. Due to the poor general conditions at the time of reintervention or due to the presence of extensive necrosis, we proceeded with a temporary cervical esophagostomy and, when feasible, a later gastrointestinal continuity restoration using colon or jejunum interposition.

Patients in the NAT group experienced a significantly higher rate of postoperative complications (*p* = 0.0266). No differences in the type or severity of complications were observed between the two groups, except for infectious complications that were significantly more frequent among NAT patients (*p* = 0.0074). Reoperation rates did not differ between SURG and NAT patients. The ICU and length of stay were similar between the two groups and no significant differences in postoperative mortality were observed. Univariate analysis for risk factors for morbidity identified neoadjuvant therapy as the only variable strongly associated with postoperative complications (OR: 2.27, 95% CI: 1.08–4.68; *p* = 0.026).

### 3.4. Pathological Data

Patients in the SURG group had a higher pT stage (*p* < 0.0001) and more advanced final pathological stages (*p* = 0.0274). No differences were observed in the total number of lymph nodes removed, the rate of positive lymph nodes, positive resection margins, or differentiation grade between the two groups. Patients in the SURG group had a higher rate of lymphovascular invasion (*p* = 0.0011).

### 3.5. Survival Analysis

**Figure 1:**
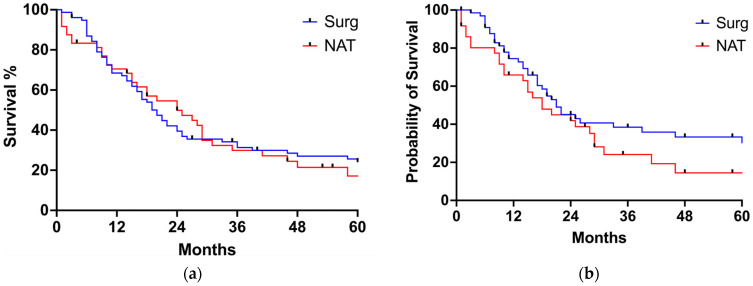
(**a**) Overall survival for the entire cohort of patients; (**b**) cancer-related survival for the entire cohort of patients.

**Figure 2:**
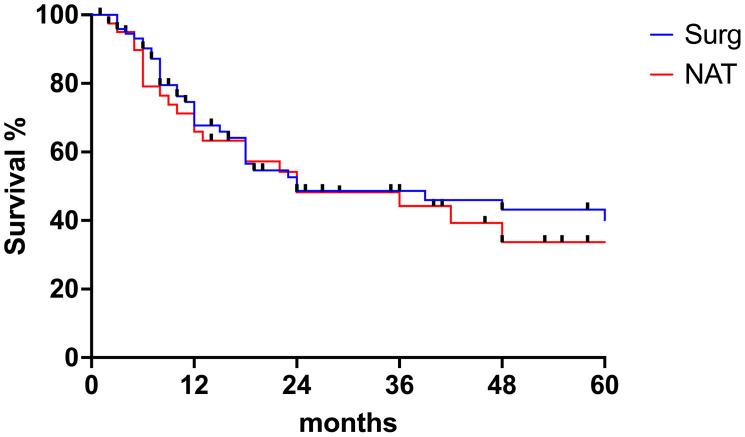
Disease-free survival for the entire cohort of patients.

**Figure 3:**
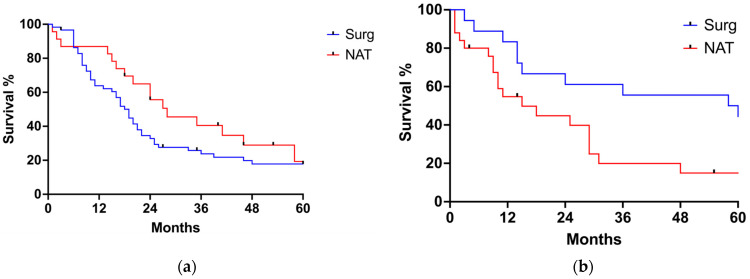
Overall survival according to histology. (**a**) EAC, (**b**) SCC.

**Figure 4:**
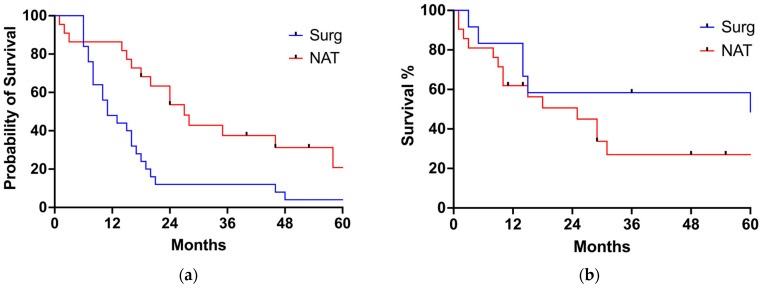
Overall survival according to histology and stage. (**a**) OS for EAC stages 3–4; (**b**) OS according to SCC stages 3–4.

The median follow-up for the entire cohort was 17 months (range 1–225 months). Twenty-nine patients (22.9%) received adjuvant treatment after surgery. Sixty patients experienced postoperative recurrence, with a median disease-free survival of 24 months (range 18–30 months). The 1 year, 3 years and 5 years survival rates for the SURG group were 68%, 31%, and 24%, respectively. The 1 year, 3 years and 5 years survival rates for the NAT group were 70%, 30%, and 17% months, respectively. There were no statistically significant differences in OS (20 months for SURG and 24 months for NAT, *p* = 0.7708) or cancer-related survival (21 months for SURG and 18 months for NAT, *p* = 0.0827) between the SURG and the NAT group (Figure 1a,b). Similarly, DFS survival was similar between the two groups (24.0 months for the SURG group, 24.0 months for the NAT group, *p* = 0.59) (Figure 2). When looking at tumor histology, the survival analysis for EAC showed a tendency towards better OS for the NAT group, but the difference did not reach statistical significance (Figure 3a). On the contrary, OS for SCC was better for the SURG group (median OS = 59 months) when compared to the NAT group (median OS = 15 months) (Figure 3b). However, considering the higher proportion of more advanced esophageal tumors in the NAT patients, we further subcategorized patients according to their initial clinical stage and compared OS in patients with stage III and IV EC receiving surgery or neoadjuvant therapy (Figure 4a,b). When considering only patients with SCC with stage III and IV, there was no difference in OS between the SURG and NAT groups. Among EAC patients with stage III-IV, the NAT group experienced a significant benefit in OS compared to the SURG group with a median survival of 46 months vs. 13 months (*p* = 0.0263). To verify any potential confounding variables that may have influenced this result, we compared EAC patients staged III-IV receiving NAT (*n* = 22) with those undergoing upfront surgery (with same histology and clinical stage) (*n* = 25), taking into account preoperative clinical and demographics data: patients receiving NAT were significantly younger (76.5 years vs. 79 years, *p* = 0.001) but there were no other differences in the preoperative clinical and demographic characteristics. Finally, we performed a multiple logistic regression adjusting for age, fitness (expressed as Charlson Comorbidity Index and ASA score), and NAT to investigate the association with 5-year OS: no variable was independently associated with the survival in our model (Table 3).

## 4. Discussion

The proportion of elderly patients with EC is expected to grow progressively in the coming years [15,16], becoming a major issue in the health system worldwide [6]. Despite this growing incidence, the ideal management of EC in older patients remains debatable [12]. Elderly patients, mostly older than 75 years, have generally been excluded or underrepresented in randomized study protocols such as the EORTC study [17,18], leading to limited literature regarding the best approach in this setting. In this retrospective study, we analyzed surgical and oncological outcomes of elderly patients with locally advanced esophageal cancer, comparing those who received upfront surgery (SURG group) with patients who received neoadjuvant treatment before surgery (NAT group). The overall postoperative morbidity and mortality rates in our cohort of patients were 48.8% and 8.7%, respectively. Although the postoperative morbidity rate aligns with previously published literature, the mortality rate is unacceptably high for a large volume esophageal center. However, it is considered tolerable given the advanced age of the patients and the high prevalence of comorbid conditions. Other studies have reported similar mortality rates in elderly patients [16]. When comparing the SURG and NAT groups, patients receiving neoadjuvant treatment experienced a significantly higher rate of postoperative complications (*p* = 0.0266). No differences in the type or severity of complications were observed between the two groups, except for infectious complications, which were significantly higher in NAT patients (0.0074). Postoperative LOS and ICU stay, as well as postoperative mortality rate, were comparable between the two groups. Univariate analysis identified neoadjuvant therapy as the only variable strongly associated with postoperative morbidity (OR: 2.27, 95% CI: 1.08–4.68; *p* = 0.026). When comparing our results with the literature, several authors have investigated the feasibility of esophagectomy in patients over 70 years of age, but very few studies have compared postoperative results of elderly patients receiving upfront surgery with those undergoing preoperative chemo- or chemoradiation followed by esophagectomy [19,20,21]. Rice et al. [19], in a retrospective study on 74 patients, compared septuagenarians who underwent esophagectomy with those who received preoperative therapy before surgery, showing similar perioperative mortality rates (0% and 3%, respectively; *p* = ns). However, patients undergoing NAT received a higher rate of perioperative blood transfusions (71.4% vs. 48.7%, *p* = 0.047) and experienced postoperative atrial arrhythmias more frequently (34% vs. 15%, *p* = 0.008). Camerlo et al. [20] presented a retrospective study in 52 patients (≥70 years) undergoing neoadjuvant therapy followed by surgery or esophagectomy alone for adenocarcinoma of the esophagus and gastric cardia: both postoperative morbidity and mortality rates were similar between the two groups. Guttmann et al. [22] conducted an observational cohort study selecting patients from the National Cancer Database (NCDB) and assessing the effect of neoadjuvant chemoradiation on overall survival and perioperative outcomes. No differences in postoperative mortality were observed, while the influence of perioperative treatment on morbidity was not analyzed. Overall, these studies indicate that a very selected group of fit elderly patients who are surgical candidates appear to tolerate neoadjuvant CRT without a significant increase in postoperative mortality, but with a possible increase in postoperative morbidity, particularly in the development of medical complications. Significant progress in the last 20 years in the perioperative management of patients undergoing esophagectomy in high-volume centers, including refinement of the surgical approach and improvements in postoperative care, allows scheduling surgery for elderly patients who received preoperative treatment after a careful preoperative evaluation [23,24]. An individualized approach is recommended to balance the therapeutic approach with the stage of the disease and the degree of comorbid conditions [16].

The actual benefit of NAT on the long-term outcomes of elderly patients undergoing esophagectomy is still to be determined. In our cohort, there was no significant difference in overall survival, cancer-related survival, and disease-free survival between the SURG and the NAT groups. Notably, overall survival was calculated from the day of surgery to death, regardless of the cause. Calculating OS from the day of surgery might underestimate survival in the NAT group. However, this is probably the most widely adopted and reproducible method among retrospective clinical studies. Moreover, the exact starting-dates of NATs are not always available in retrospective datasets, especially since many patients are referred from other centers, therefore this would have resulted in having too many missing data. Finally, using the NAT starting-date might also introduce a bias inherent to the length of the different treatment regimens and eventual delays in referring patients to surgery. When categorizing patients according to tumor histology and stage, patients with EAC (stage III-IV) receiving NAT experienced a survival benefit compared to the SURG group. This was not true for patients with SCC with stage III and IV tumors, whose OS did not differ between the two groups. The current literature shows discordant findings. Guttman et al. [22] reported that neoadjuvant chemoradiation was associated with improved overall survival (HR = 0.76, 95% CI [0.70–0.82], *p* ≤ 0.001) compared to esophagectomy alone. In contrast, Camerlo et al. [20] showed no significant differences in OS and DSF between patients undergoing surgery alone and those receiving NAT before surgery (30 months vs. 23 months, *p* = 0.304 and 21 vs. 22 months, *p* = 0.807, respectively). It is difficult to compare the different outcomes reported by different authors due to variation in histology, tumor stage, and surgical approach. In our study, including both EAC and SCC tumors, different results were obtained by subcategorizing patients according to histology. While EAC patients experienced a survival benefit when receiving NAT prior to surgery, SCC patients did not. OS for SCC was better for the SURG group (median OS = 59 months) when compared to the NAT group (median OS = 15 months) (Figure 3b). However, the SCC group included more patients with earlier stages, as well as a higher number of patients who received upfront esophagectomy.

Elderly patients have unique characteristics that require careful consideration, including life expectancy, concurrent comorbidities and functional status, risk of treatment-related morbidity, and desire to receive therapy [8]. Because of their increased frailty, elderly patients may receive less aggressive treatment compared to younger patients, which may affect their overall survival [15,16]. The high prevalence of comorbidities, the higher rate of postoperative morbidity, and, in general, the reduced life expectancy, may lower the potential survival benefit of the multimodal approach in elderly people [25].

Surgical oncology in elderly people introduces several challenges. The decline in physiological systems and the presence of comorbidities impact surgical management and tolerance to oncological treatments. The toxicity of chemotherapy and/or radiotherapy regimens may be significant in geriatric patients and limited physiological reserve could undermine the chance to complete multimodal oncological treatments. Neoadjuvant chemotherapy may weaken patients and compromise their functional reserve before surgery. To address these concerns and explore alternative risk-mitigation strategies, several approaches can be considered. The main scope of preoperative assessment in the elderly population should be identifying frail patients. A preoperative geriatric evaluation can help assess the vulnerability of older patients and should include assessment of cognitive, functional, somatic, nutritional and social domains [26]. Combining information from the geriatric assessment with cancer-related information, including treatment options and expected outcomes, may optimize shared decision-making. Frail and comorbid patients should be directed towards medical optimization and prehabilitation before surgery [27]. Personalized perioperative care pathways tailored to the specific needs of frail patients may help minimize postoperative complications and facilitate faster recovery. Exploring novel treatment approaches, such as targeted therapies or immunotherapies, which may exhibit a more favorable toxicity profile compared to traditional chemotherapy, could offer promising avenues in reducing postoperative morbidity in this patient population [28]. Assessments of frailty, multimorbidity, and patients’ preference towards treatment options are essential in the decision-making process of geriatric patients with cancer. Some authors have suggested implementing an oncogeriatric care pathway in standard care for all cancer patients aged 70 years or older [29]. This pathway combines a geriatric assessment with the exploration of patients’ expectations and integrates geriatric interventions into cancer care. This approach could improve not only treatment outcomes (including reduction in toxicity) but also the postoperative quality of life (QOL). In addition to commonly studied clinical outcomes such as complications and survival, patient-reported outcomes, such as loss of independence and QOL, are of great importance to older patients and should be considered when tailoring the therapeutic approach for elderly cancer patients. By integrating geriatric assessments and considering patient-centered outcomes, healthcare providers can better inform treatment decisions and enhance overall care for elderly patients with esophageal cancer.

Our study has several limitations. It is a retrospective observational study that includes only patients eligible for surgery and patients with different tumor stages and histology. These factors, along with some missing data, represent a potential selection bias that must be considered when analyzing our results. However, it represents the experience of a high-volume center for esophageal surgery and one of the few studies focusing on the comparison of elderly patients receiving multimodal treatment versus surgery alone. Further studies are needed to better elucidate the optimal approach in elderly patients with esophageal cancer. Prospective multicenter studies with standardized treatment protocols and homogeneous patient populations could minimize biases and provide more robust evidence on this topic.

## 5. Conclusions

In conclusion, for patients 75 years and older with esophageal cancer, neoadjuvant chemoradiation prior to esophagectomy is associated with a higher rate of postoperative complications compared to esophagectomy alone, with an equal rate of postoperative mortality. The two approaches showed similar long-term outcomes, except for patients with stage III-IV EAC, who experienced a survival benefit after NAT. Considering the unique characteristics of elderly patients, the choice of a multimodal approach should be tailored to each case in a multidisciplinary setting and balanced with the potential higher risk of postoperative complications, potential toxicity related to chemoradiation, and reduced life expectancy. While multimodal treatment seems reasonable for selected fit patients with more advanced clinical stages, an upfront surgery approach can be safely and effectively considered for earlier stages of the disease. The inclusion of elderly patients in future randomized trials could help clinicians identify those most suitable for trimodal therapy and those more likely to experience a survival benefit through a multimodal approach, further characterizing optimal care for older patients with esophageal cancer.

## Figures and Tables

**Table 1:** Demographic data of patients undergoing upfront surgery (SURG) or neoadjuvant therapy (NAT).

Characteristics	SURG (*n* = 78)	NAT (*n* = 49)	*p*
Sex (*n*, %)			
Male	54, 69.2%	34, 69.4%	ns
Female	24, 30.8%	15, 30.6%	
Age median (IQR)	79 (77–82)	77 (76–79)	0.0038
Preoperative albumin level; median (IQR)	38 (34–41)	41 (39–44)	ns
Preoperative prealbumin level; median (IQR)	237 (191–276)	222 (187–252)	ns
Preoperative transferrin level; median (IQR)	2.36 (2.02–2.66)	2.56 (2.21–2.88)	ns
BMI (kg/m^2^) median (IQR)	25.2 (22.8–27.6)	24.2 (21.3–27.2)	ns
Charlson index; median (IQR)	6 (6–7)	6 (5–7)	ns
Comorbidities (*n*, %)			
Diabetes	15, 19.23%	4, 8.16%	ns
Cardiac disease	34, 43.59%	14, 28.57%	ns
Hypertension	42, 53.85%	25, 51.02%	ns
COPD ^1^	18, 23.08%	5, 10.20%	ns
Peripheral vascular disease	13, 16.67%	10, 20.41%	ns
Pre-existing esophageal disorder	16, 20.51%	6, 12.24%	ns
Renal insufficiency	6, 7.69%	3, 6.12%	ns
History of malignancies	14, 17.95%	10, 20.41%	ns
Alcohol	20, 28.17%	8, 17.39%	ns
Smoke	16, 22.54%	6, 13.04%	ns
ECOG status (*n*, %)			
0	48, 61.5%	25, 51.0%	ns
1	28, 35.9%	23, 46.9%	ns
2	2, 2.56%	1, 2.04%	ns
Karnofsky scale			
50–60	0, 0.0%	1, 2.04%	ns
70–80	17, 21.8%	11, 22.45%	
90–100	61, 78.2%	37, 75.51%	
ASA ^2^ score			
ASA 1	1, 1.28%	0, 0.0%	0.0371
ASA 2	18, 23.08%	23, 46.94%	
ASA 3	55, 70.51%	25, 51.02%	
ASA 4	4, 5.13%	1, 2.04%	
Histology			
Adenocarcinoma (EAC)	60, 76.92%	23, 46.94%	0.0005
Squamous Cell Carcinoma (SCC)	18, 23.08%	26, 53.06%	
Initial clinical stage			
Stage I	0, 0.0%	0, 0.0%	<0.0001
Stage II	40, 51.28%	7, 14.29%	
Stage III	36, 46.15%	38, 77.55%	
Stage IV	2, 2.57%	4, 8.16%	

^1^ COPD = Chronic obstructive pulmonary disease, ^2^ ASA = American Society of Anesthesiologists.

**Table 2:** Surgical results: comparisons of surgical outcomes of the SURG group vs. the NAT group.

Variable (*n*, %)	SURG (*n* = 78)	NAT (*n* = 49)	*p*
Surgical procedure			
Ivor-Lewis	60, 76.9%	44, 89.8%	ns
McKeown	7, 9.0%	3, 6.1%	
Other	11, 14.1%	2, 4.1%	
Postoperative morbidity rate	32, 41.0%	30, 61.2%	0.0266
Type of complications			
Anastomotic leak	4, 5.1%	6, 12.2%	ns
Type I	0, 0.0%	1, 16.7%	
Type II	2, 50.0%	2, 33.3%	
Type III	2, 50.0%	3, 50.0%	
Conduit necrosis	1, 1.3%	1, 2.0%	ns
Type I	0, 0.0%	0, 0.0%	
Type II	0, 0.0%	0, 0.0%	
Type III	1, 100%	1, 100%	
Chyle leak	0, 0.0%	1, 2.0%	ns
Type I	0, 0.0%	0, 0.0%	
Type II	0, 0.0%	0, 0.0%	
Type III	0, 0.0%	1, 100%	
Recurrent laryngeal nerve palsy	2, 2.6%	2, 4.1%	ns
Type I	2, 100%	2, 100%	
Type II	0, 0.0%	0, 0.0%	
Type III	0, 0.0%	0, 0.0%	
Pulmonary	11, 14.1%	13, 26.5%	ns
Cardiac	9, 11.5%	9, 18.4%	ns
Gastrointestinal	8, 10.2%	7, 14.3%	ns
Urological	1, 1.3%	1, 2.0%	ns
Neurologic	3, 3.8%	5, 10.2%	ns
Thromboembolic	4, 5.1%	1, 2.0%	ns
Infection	4, 5.1%	10, 20.4%	0.0074
Other	3, 3.8%	2, 4.1%	ns
Late complications	16, 20.5%	12, 24.5%	ns
ICU ^1^ stay (days); median (IQR)	2 (2–4)	2 (2–4)	ns
Length of stay (days); median (IQR)	15 (12–19)	14 (11–19)	ns
Need for reoperation	6, 7.7%	6, 12.2%	ns
Postoperative mortality	4, 5.1%	7, 14.6%	ns

^1^ ICU = Intensive Care Unit.

**Table 3:** Multiple logistic regression analyzing the correlation among age, fitness (expressed as Charlson Comorbidity Index and ASA score), NAT and 5-year OS.

	Univariate Analysis		Multivariate Analysis	
Variable	Odds Ratio (95% CI)	*p*	Odds Ratio (95% CI)	*p*
Age > 85 years	2.0 (0.2–17)	0.52	2.9 (0.5–58)	0.33
Sex (M:F)	0.7 (0.3–1.7)	0.42	1.5 (0.6–3.9)	0.37
CCI * > 5	0.6 (0.2–1.8)	0.37	0.7 (0.2–2.0)	0.52
ASA ^§^ score > 2	0.5 (0.1–1.4)	0.16	0.5 (0.1–1.5)	0.24
NAT °	1.6 (0.6–4.3)	0.33	1.5 (0.6–4.3)	0.43

* Charlson Comorbidity Index, ^§^ American Society of Anestesiology, ° neoadjuvant treatment.

## Data Availability

The data presented in this study are available on request from the corresponding author.
